# Genome-Wide Investigation of *MADS-Box* Genes in Flower Development and Environmental Acclimation of *Lumnitzera littorea* (Jack) Voigt

**DOI:** 10.3390/ijms26041680

**Published:** 2025-02-16

**Authors:** Linbi Zhang, Yuchen Yang, Ying Zhang, Fusun Yang

**Affiliations:** 1 School of Tropical Agriculture and Forestry, Hainan University, Haikou 570228, China; zlinbi@163.com; 2 School of Ecology, Sun Yat-sen University, Shenzhen 518107, China; yangych68@mail.sysu.edu.cn; 3 School of Life Science and Technology, Lingnan Normal University, Zhanjiang 524048, China

**Keywords:** transcription factor, endangered mangrove species, flower morphology, cold acclimation

## Abstract

*Lumnitzera littorea* (Jack) Voigt is an endangered mangrove species in China. Low fecundity and environmental pressure are supposed to be key factors limiting the population expansion of *L. littorea*. Transcription factors with the MADS-box domain are crucial regulators of plant flower development, reproduction, and stress response. In this study, we performed a comprehensive investigation into the features and functions of *MADS-box* genes of *L. littorea*. Sixty-three *LlMADS* genes with similar structure and motif composition were identified in the *L. littorea* genome, and these genes were unevenly distributed on the 11 chromosomes. Segmental duplication was suggested to make a main contribution to the expansion of the *LlMADS* gene family. Some *LIMADS* genes exhibited differential expression in different flower types or in response to cold stress. Overexpression of the B-class gene *LlMADS37* had substantial effects on the flower morphology and flowering time of transgenic *Arabidopsis* plants, demonstrating its key role in regulating flower morphogenesis and inflorescence. These findings largely enrich our understanding of the functional importance of *MADS-box* genes in the inflorescence and stress acclimation of *L. littorea* and provide valuable resources for future genetic research to improve the conservation of this species.

## 1. Introduction

*Lumnitzera littorea* (Jack) Voigt., a typical true mangrove species of family Combretaceae, is sparsely distributed in intertidal swamps of the Indo-West Pacific region, such as India, Sri Lanka, Indonesia, Malaysia, and China [1,2]. In terms of ecology, *L. littorea* plays an important role in carbon storage, coastal protection, and nutrient filtering [3,4,5]. Moreover, *L. littorea* also has high economic and medicinal value. The wood of *L. littorea* can be used as building material or fuel [6], and the leaves are rich in secondary metabolites with antibacterial, anti-inflammatory, and anticancer properties, such as alkaloids, steroids, triterpenoids, flavonoids, and ellagic acid, which can be used for therapy against bacterial infection, diabetes, and nerve injury [7,8,9,10,11,12]. However, in China, the population size of *L. littorea* is extremely small, and it has been listed in Category I of the National Key Protected Wild Plants [13,14]. One of the reasons for the small number of *L. littorea* is its low fecundity. Our previous study showed that the embryo abortion rate of *L. littorea* reaches up to 76%, which largely restricts the natural regeneration and dispersal of *L. littorea* [15]. Plant reproduction highly depends on correct programming of flower development [16,17]. For *L. littorea*, there are two types of flowers: the stigmas of ~60% of flowers stretch out of the buds before blooming, while the other 40% are covered by petals before flowering [18].

Plant flower development and inflorescence are under the regulation of a multitude of genes. Of them, transcription factors (TFs), in particular MADS-box TFs, play a crucial role in modulating the programming of flower development [19]. MADS-box TFs exist in most eukaryotes including plants, animals, and fungi [20]. All the TFs in this family contain a conserved DNA-binding motif (CArG-box (CC(A/T)_6_GG) with an NAA extension), and they are named after four of the founding members: MINICHROMOSOME MAINTENANCE FACTOR1 (MCM1) from *Saccharomyces cerevisiae*, AGAMOUS (AG) from *Arabidopsis thaliana*, DEFICIENS (DEF) from *Antirrhinum majus*, and SERUM RESPONSE FACTOR (SRF) from *Homo sapiens* [16,21,22,23]. *MADS-box* genes are categorized into two distinct clades according to the conserved domains they have: Type I/M-type *MADS-box* genes that consist of an SRF-like domain and Type II/MIKC-type *MADS-box* genes that contain an MEF2-like MADS domain, an intervening domain, a keratin-like domain (K-box), and a highly variable C-terminal region [24,25,26]. Type I *MADS-box* genes are further divided into three subgroups, Mα, Mβ, and Mγ [27], and Type II genes can be divided into two major subgroups, MIKC* and MIKCC, depending on the structures of intervening and K-box domains [28].

*MADS-box* genes have been reported to play essential roles in plant growth and development, such as floral organogenesis and seed development [29]. Some MADS-box TFs are involved in the modulation of the ABCDE model for the formation and development of floral organs. The TFs of class A coordinate the specification of sepals and petals, and those of class B, C, and E are required for stamen specification [24,30,31,32]. Changes in these genes and their product proteins will lead to developmental defects in floral organs [33,34]. In *Arabidopsis*, the class A gene *AP1* mediates the suppression of bracts during flower development, and mutations in *AP1* lead to a phenotypic transformation from whorl organs into bract-like structures [35,36]. In another instance, loss of function of *SEPALLATA* (*SEP*) genes, *SEP1*, *2*, and *3*, in *Arabidopsis* resulted in a conversion of all other flower organs into sepals [37]. In our previous study, we investigated the role of *MADS-box* genes that were identified in *L. littorea* using *de novo* assembled transcriptome data, and these genes exhibited differential expression between the two types of flowers (stigmas covered by petals or not before flowering), indicating that *MADS-box* genes play an important role in regulating flower development in *L. littorea* [18]. However, transcriptome assembly represents the genes currently being expressed, thus leading to an underestimation of the *MADS-box* genes in *L. littorea*. An identification and characterization of the *MADS-box* genes with the help of whole-genome sequencing would present a more comprehensive picture of their features and biological functions in the inflorescence and flower development of *L. littorea*.

In addition, *MADS-box* genes have been reported to be involved in the abiotic stress response [38,39]. In rice (*Oryza sativa* Indica), seven *MADS-box* genes display varying expression levels when exposed to cold, salt, and/or drought stress, with three of them being downregulated in response to dehydration and salt stress [40]. In another instance, *MADS-box* genes in barley (*Hordeum vulgare*) were also reported to be involved in stress responses, including salt and waterlogging stress [39]. *L. littorea* lives in a harsh environment with high salt content, tidal flooding, and hypoxia, and its growth, development and reproduction are sensitive to environmental fluctuations [41,42,43]. For example, in 2008, *L. littorea* trees in Hainan Dongzhai Harbor were all frozen to death due to the extremely low temperature. Our previous study revealed that genes associated with cold sensing and stress responses were substantially upregulated in leaves of *L. littorea* seedlings to cope with exposure to cold stress [43]. However, none of the current studies pay attention to the role of *MADS-box* genes in stress responses in *L. littorea;* thus, an investigation on the expression changes of *MADS-box* genes upon exposure to stress conditions may provide new insights into the regulatory mechanisms underlying the acclimation of *L. littorea* to environmental alterations.

In this study, 63 *MADS-box* genes were identified in the genome of *L. littorea*. The expression profiles of these genes were then characterized, and the key *MADS-box* genes associated with the flower development and stress responses of *L. littorea* were identified. Overexpression of one *MADS-box* gene, *LlMADS37*, in transgenic Arabidopsis further confirmed its biological importance in flower morphogenesis and flowering time. Our findings provide a research basis for future in-depth investigations on the molecular function of *MADS-box* genes of *L. littorea*.

## 2. Results

### 2.1. Identification of MADS-Box Genes in L. littorea

In the genome of *L. littorea*, a total of 71 genes were identified with the MADS-box domain. Of them, eight genes were supposed to give rise to truncated product proteins with only a small number of amino acids, indicative of the appearance of premature termination codons or alignment errors; thus, these genes were excluded from downstream analysis. The retained 63 *MADS-box* genes (denoted as *LIMADS* genes hereafter) were then renamed according to their positions on the chromosome. The length of protein sequences encoded by these *LlMADS* genes ranged from 103 (*LlMADS34*) to 412 (*LlMADS63*) amino acids, where 41 product proteins (accounting for 63.49%) consist of 200–300 amino acids (see Table S1 for details). The molecular weight (MW) of these LlMADS proteins ranged from 12.11 KDa to 46.65 KDa, and the isoelectric point (pI) was 4.26–10.63; 44 proteins (accounting for 69.84%) had a pI greater than 7.

### 2.2. Classification and Phylogenetic Analysis

**Figure 1:**
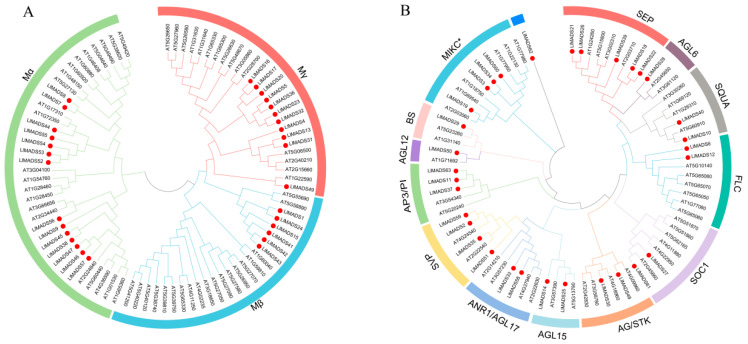
Phylogenetic trees of *MADS-box* genes of *L. littorea* and *Arabidopsis*. (**A**) Phylogenetic tree of Type I *MADS-box* genes of *L. littorea* and *Arabidopsis*. The three subgroups Mα, Mβ and Mγ are marked with different colors. (**B**) Phylogenetic tree of Type II *MADS-box* genes of *L. littorea* and *Arabidopsis*. The 14 subgroups of Type II *MADS-box* genes are marked with different colors. For both panels, *MADS-box* genes of *L. littorea* are highlighted by red circles.

*LlMADS* genes were also clustered into two clades, Type I (31 genes) and Type II (32 genes), according to the sequence and domain similarities to the homologs of *Arabidopsis*. Phylogenetic analysis of Type I of *LlMADS* and *Arabidopsis MADS-box* genes further identified three subclades, Mα, Mβ, and Mγ, which consist of 14, 6, and 11 members, respectively (Figure 1A). Type II *LlMADS* genes, except *LlMADS62*, fell into two subclades, MIKCC (28 members) and MIKC* (4 members), where MIKCC-type proteins were further divided into 12 subgroups, BS, SVP, SQUA (AP1/FUL, class A), AP3/PI (class B), AG/STK (class C and D), SEP (class E), SOC1, FLC, AGL6, AGL12, AGL15, and ANR1/AGL17 (Figure 1B). The SEP subgroup contained the maximum number of genes (five), followed by SVP (four) and the others (one to three members). *LlMADS62* gene alone formed a cluster at the base of the phylogenetic tree without any homologous detected in *Arabidopsis*, indicating its relatively early origin in the gene family and different evolutionary history.

### 2.3. Chromosomal Locations and Collinearity Analysis

**Figure 2:**
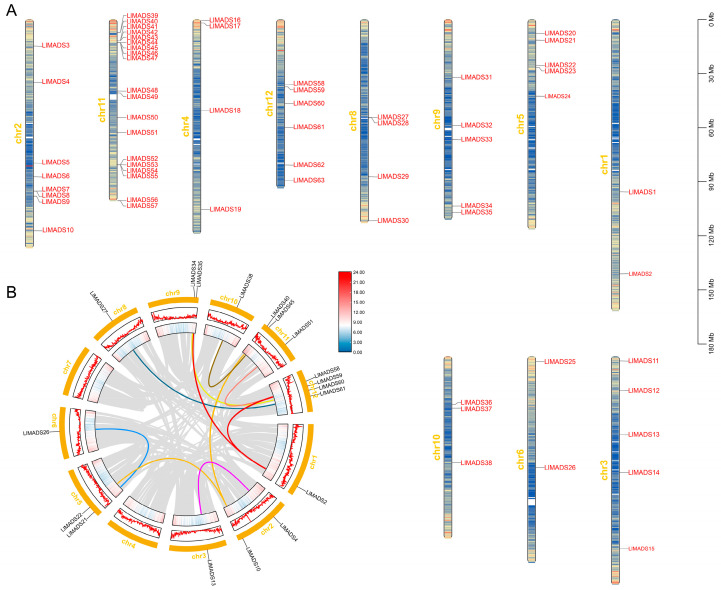
Chromosomal locations and collinear relation of *LlMADS* genes. (**A**) Locations of 63 *LlMADS* genes on the 11 *L. littorea* chromosomes. (**B**) Collinear relation of 63 *LlMADS* genes Gray lines represented all co-localized blocks in *L. littorea* genome, and those related to *LlMADS* genes are highlighted in other colors.

The locations of the 63 *LlMADS* genes on *L. littorea* chromosomes are shown in Figure 2A. The results revealed an uneven distribution of *LlMADS* genes on the 11 chromosomes; chr11 consists of the largest number of *LlMADS* genes (19), while no *LlMADS* gene was detected on chr7. Interestingly, 14 out of the 19 *LlMADS* genes on chr11 were from the Type I clade, while the other 5 were from the ABCDE model and SVP subfamilies of the Type II clade. Other chromosomes contained 2–8 *LlMADS* genes.

Gene duplication relationships among *LlMADS* genes were inferred by collinearity analysis. Ten linear pairs of fifteen *LlMADS* genes were identified in *L. littorea* (Figure 2B). It is worth noting that none of the collinearity events occurred on the same chromosome, highlighting the roles of chromosome segment duplications in the expansion of the *MADS-box* gene family in *L. littorea*.

### 2.4. Conserved Motif and Gene Structure Analysis

**Figure 3:**
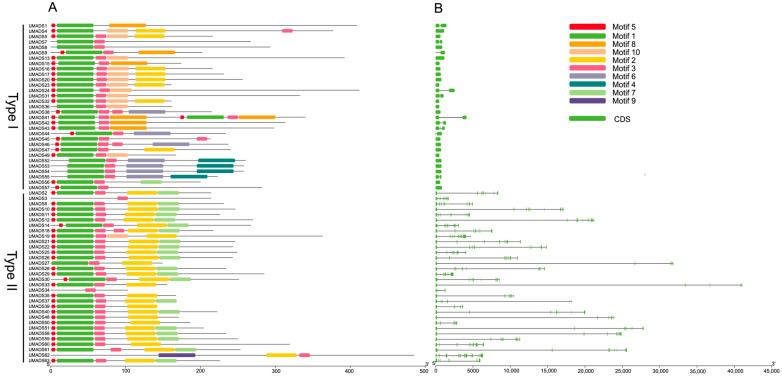
Conserved motifs and gene structure of *LIMADS* genes. (**A**) Conserved motifs contained in each *LIMADS* gene. Different motifs are represented by different colors. (**B**) Gene structure of each *LIMADS* gene. Exons are represented by green boxes and introns are represented by gray lines.

A total of ten conserved motifs were found in LlMADS proteins (Figure 3A). Each LlMADS protein contained one to eight motifs. Motif1, 3, and 5 were the most common domains that were shared by most LlMADS proteins at the N-terminus regions. In contrast, Motif 7, which is a K-box domain, was only found in Type II LlMADS proteins. In contrast to conserved N-terminus regions, C-terminus regions of LlMADS proteins were of relatively high variability.

Changes in exon/intron numbers are important for genes and can lead to different splicing variants and protein products. For *LlMADS* genes, the number of introns ranged from zero to twelve (Figure 3B). Moreover, intron numbers and sizes were substantially different between Type I and Type II *LlMADS* genes. The majority of Type I genes contained either one or no introns, while Type II genes had multiple and larger introns, especially *LlMADS27* and *LlMADS33*, which had extremely longer introns compared to the others.

### 2.5. Cis-Regulatory Element Prediction

**Figure 4:**
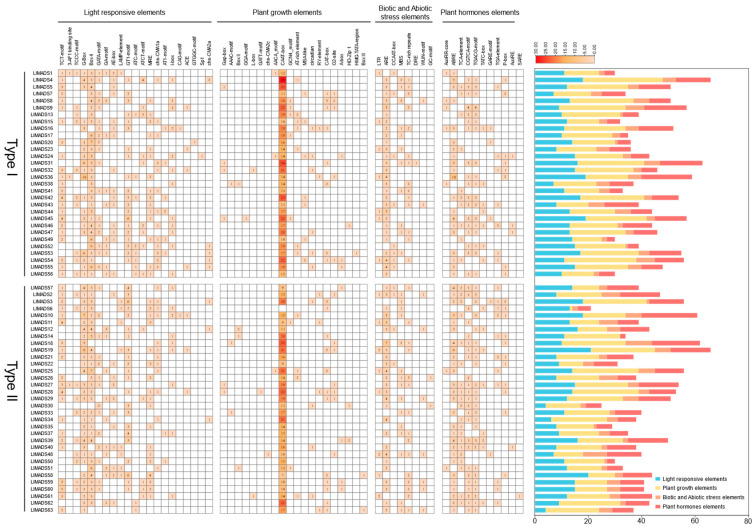
Putative *cis*-regulatory elements in promoter regions (2000 bp upstream of transcription start site) of *LlMADS* genes.

Sixty *cis*-regulatory elements were identified in promoter regions of *LIMADS* genes and classified into four classes according to their regulatory functions, including light-responsive, plant growth and development, biotic and abiotic stress, and plant hormone-responsive elements (Figure 4). Plant growth and development-related elements were of the highest abundance, which account for 45% of all elements, followed by light-responsive elements (31%), phytohormone-responsive elements (14%), and biotic and abiotic stress-responsive elements (10%). Eleven members were detected in the phytohormone-responsive class, which are associated with the response to auxin (AuxRR-core, AuxRE, and TGA-element), methyl jasmonate (MeJA) (CGTCA motif and TGACG motif), salicylic acid (SA) (TCA-element and SARE), abscisic acid (ABA) (ABRE), and gibberellin (GA) (TATC-box, P-box and GARE motif). Among them, ABRE elements, CGTCA motif, and TGACG motif were the most prevalent in *LlMADS* genes, existing in 47, 42, and 43 *LlMADS* genes, respectively. These indicated that the majority of *LlMADS* genes may be under the regulation of ABA and MeJA. In the class of biotic and abiotic stress-responsive elements, ARE was present with the highest frequency; a total of 138 AREs were identified in 86% of the *LlMADS* genes (54/63).

### 2.6. Expression Profiles of LlMADS Genes Involved in Flower Development

**Figure 5:**
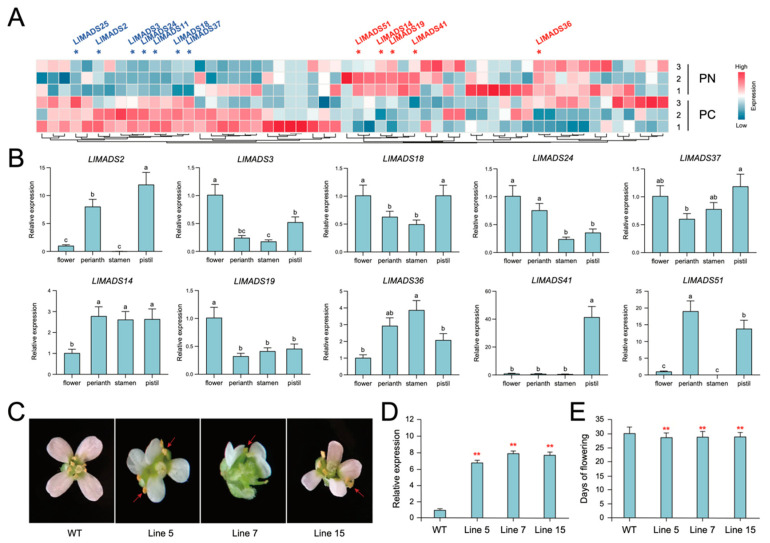
Expression profiles of *LlMADS* genes between different types and parts of *L. littorea* flowers and flower morphology changes in *Arabidopsis* upon overexpression of *LlMADS37*. (**A**) Heatmap illustrating expression profiles of *LlMADS* genes between two types of *L. littorea* flowers: flowers with stigmas covered by petals before blooming (PC) and those not covered by petals (PN). (**B**) Expression profiles of 10 *LlMADS* genes in different parts of *L. littorea* flower by qRT-PCR. Different lowercase letters represent significantly statistical differences (*p*-value < 0.05) in expression levels by ordinary one-way ANOVA. (**C**) Flower phenotypes of wild-type (WT) and *OX-LlMADS37* transgenic lines of *Arabidopsis*. Red arrows mark the morphological changes in flowers (**D**) Relative expression of *LlMADS37* in WT and *OX-LlMADS37* transgenic lines by qRT-PCR. (**E**) Days from germination to flowering in WT and *OX-LlMADS37* transgenic lines. For panels (**D**,**E**), ** represents significant difference between transgenic lines and WT at *p*-value < 0.01 level (Wilcoxon test).

The expression profiles of *LlMADS* genes in two types of *L. littorea* flowers were characterized using transcriptome data. Two Type I genes, *LlMADS36* and *41*, and three MIKCC-type genes, *LlMADS14*, *19*, and *51*, were found to be substantially more expressed in the flowers whose stigmas are not covered by petals before blooming (PN) than those with stigmas covered by petals (PC), whereas seven genes (*LlMADS2*, *3*, *11*, *18*, *24*, *25*, and *37*) were overexpressed in PC (Figure 5A). These are largely consistent with our previous qRT-PCR results [18]. Moreover, the weighted gene co-expression network analysis (WGCNA) based on the transcriptome data revealed that *LlMADS11*, *18*, *25*, and *37* were positively correlated with the phenotype of PC (Table S3). Of them, *LlMADS37* showed the highest correlation, implying its important role in regulating the flower morphology of *L. littorea*. In contrast, *LlMADS19* and *36* were significantly positively related to PN (Table S4). We further investigated their expression in different parts of *L. littorea* flowers. Compared to the whole flower, *LlMADS2*, *14*, and *51* exhibited significantly higher expression, while *LlMADS3*, *18*, and *19* were expressed at relatively low levels (Figure 5B). Genes *LlMADS14* and *36* were overexpressed in the stamen, and *LlMADS2*, *14*, *41*, and *51* were highly expressed in the pistil.

*LlMADS37* overexpression transgenic lines of *Arabidopsis* (*OX-LlMADS37*) were established to investigate the functions of *LlMADS37* in floral morphogenesis (Figure S1). Three *OX-LlMADS37* T3 transgenic lines with confirmed high expression of *LlMADS37* and morphological change were selected for the experiments (Figure 5C,D). All three *OX-LlMADS37* lines exhibited abnormal flower morphology, including asymmetric petals, incomplete stamens, and heterotopic filaments (Figure 5C). Moreover, compared to the wild type (WT), the *OX-LlMADS37* transgenic lines displayed earlier flowering by approximately 2–3 days (Figure 5E), highlighting their key functional role in regulating the normal florescence of plants.

### 2.7. Expression Profiles of LlMADS Genes Under Cold and Salt Stress

**Figure 6:**
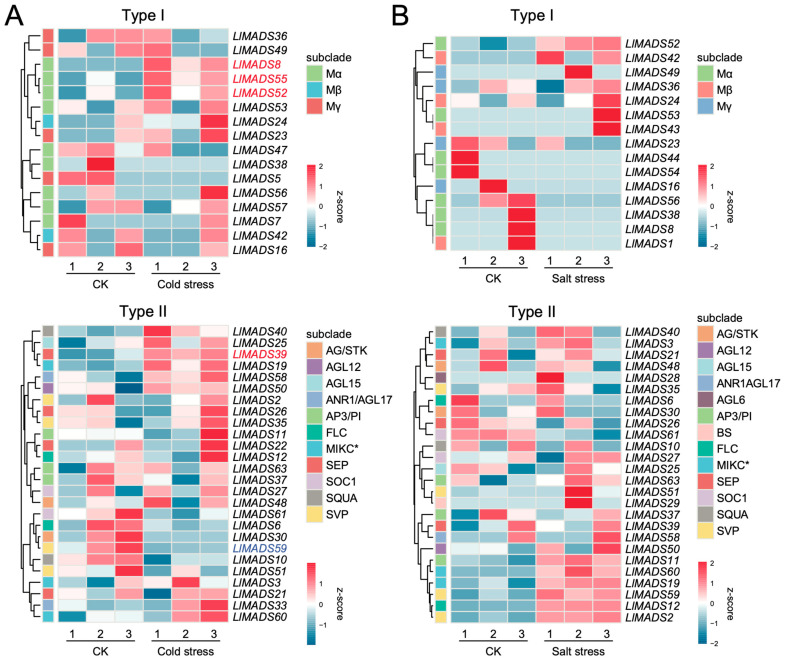
Expression profiles of *LlMADS* genes in response to cold and hyper-salinity stress. (**A**) Heatmap illustrating expression profiles of Type I (upper) and II *LlMADS* genes (lower) in plants grown under control (CK) and cold stress condition. (**B**) Heatmap showing expression levels of Type I (upper) and II *LlMADS* genes (lower) upon CK and hyper-salinity stress.

When subjected to cold treatment, three Type I *LlMADS* genes (*LlMADS8*, *52*, and *55*) and one Type II gene (*LlMADS39*) were significantly upregulated in *L. littorea* seedlings (Figure 6A), which may be associated with the responses of *L. littorea* to low ambient temperature. Of them, *LlMADS39* was further confirmed by WGCNA to play a positive role in responding to c stress (correlation coefficient = 0.95). *LlMADS39* belonged to the module “green”, and the genes in this module were significantly enriched in peroxidase activity, oxidative stress response, DNA repair, and the metabolic processes of carboxylic acid and nitrogen compounds (Figure S1). In contrast, *LlMADS59* of Type II was downregulated (Figure 6A). When exposed to salt stress, no *LlMADS* genes showed significantly differential expression (Figure 6B), suggesting a weak association between *MADS-box* genes and hyper-salinity responses in *L. littorea*.

## 3. Discussion

*L. littorea* is a rare mangrove species in China, with only few wild individuals in Sanya Tielu harbor and Lingshui Dadun village of Hainan Island. A comprehensive understanding of the molecular mechanisms underlying the reproductive and environmental acclimation of *L. littorea* can aid in its conservation and restoration. MADS-box TFs have been reported to play key roles in regulating the expression of genes related to growth, development, reproduction, and responses to abiotic stresses of plants, such as rice [44], bread wheat (*Triticum aestivum*) [45], potato (*Solanum tuberosum*) [46], *A. thaliana* [24,27], and mangrove species *Rhizophora apiculata* and *Kandelia obovata* [47]. In this study, we identified 63 *MADS-box* genes in *L. littorea*, which is similar to *R. apiculata* (65 genes) [47], but much less than some terrestrial plants, such as *A. thaliana* (107 genes), *S. lycopersicum* (131 genes) [48], and *P. trichocarpa* (105 genes) [49]. Both tandem and segmental duplication in the genome can contribute to the emergence of new members of a gene family [50]. In *L. littorea*, 10 segmental duplication events involving 15 *LIMADS* genes were identified; on the contrary, no tandem duplication was detected (Figure 2B), indicative of the major role of segmental duplication in the expansion of the *MADS-box* gene family in *L. littorea*. Generally, the emergence of novel gene members provides plants with raw materials for the potential development of new gene functions, and thus is important for plants’ genetic innovation and evolution [51]. However, mangroves have been found to evolve with a significant loss of genes, especially in gene families with large members [52]. Such a reduction in gene number may help mangrove plants to stabilize their gene regulatory network and improve their adaptive capacities to harsh intertidal environments.

**Figure 7:**
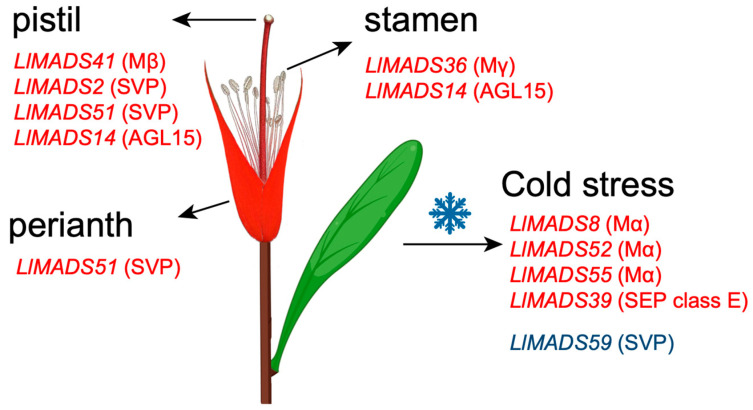
Schematic figure illustrating the *LlMADS* genes associated with flower development and stress response of *L. littorea*. *LlMADS* genes of upregulation are presented in red, while those of downregulation are presented in blue. Subgroups that these genes belong to are presented in parentheses.

Similar to other plants, MADS-box TFs in *L. littorea* are also classified into two major clusters, Type I (M-type) and Type II (MIKC-type), according to the conserved domains they have [53]. Type II genes are further divided into 13 subgroups. Of them, model B (*AP3*/*PI*) genes have been reported to play important roles in petal and stamen formation [54]. In *L. littorea*, all three class B genes (*LlMADS11*, *LlMADS37*, and *LlMADS63*) belong to the AP3 lineage, and no PI genes were observed (Figure 1). *AP3* and *PI* genes are mainly different in their sequences at the C-terminal [55,56]. Figure 7 summarizes the *LlMADS* genes that are highly expressed in the main parts of *L. littorea* flowers. Overexpression of *LlMADS37* in *Arabidopsis* transgenic lines further confirmed that transcriptional changes in *LlMADS* genes have substantially negative impacts on flower morphology, including asymmetric petals, incomplete stamens, and heterotopic filaments (Figure 5C), demonstrating their key role in modulating petal and stamen development. Similarly, heterologous overexpression of *AP3* of *Areca catechu* led to an increase in the number of flower petals of *Arabidopsis* plants [57], while overexpression of *AP3* in soybean resulted in flag petals with a more compact and denser diameter than those in the wild type [58]. Moreover, compared to WT, overexpression of *LlMADS37* also induced earlier flowering of *Arabidopsis* plants by approximately 2–3 days (Figure 5E). Together, our findings highlighted the key functions of model B genes in regulating the reproductive process of *L. littorea*.

The specific binding of TFs and CREs in gene promoter regions plays an important role in the regulation of gene transcription [59]. In this study, we identified a large number of CREs associated with plant growth and hormone responses in the promoter region of the *LIMADS* genes (Figure 4). Almost all the *LIMADS* genes, except for *LIMADS17*, contain one to eight hormone-related elements, which indicated that the expression of *LIMADS* genes is largely under the regulation of phytohormones. Of them, ABRE is of the highest abundance (present in 86% of *LIMADS* genes), followed by CGTCA and TGACG motifs (Figure 4). ABRE plays a vital role in activating ABA signaling, while the CGTCA motif and TGACG motif are related to MeJA responsiveness [60,61]. Both ABA and JAs have been reported to possess important biological significance for plant flower development and inflorescence [62,63]. In *Arabidopsis*, ABA was supposed to be involved in photoperiodic response, and deficiency in ABA biosynthesis caused late flowering [62]. Similarly, Negin et al. [64] also showed that inhibition of ABA signaling led to early flowering and smaller flowers in *Arabidopsis*. In rice, JA was shown to participate in the development of rice spikelet via regulating the expression of three *MADS-box* genes of model E genes [63]. Different from *LlMADS11* and *63*, *LlMADS37* was found to be under the regulation of SA and GA as well (Figure 4). In *Arabidopsis*, GA triggers the degradation of DELLA protein, which activates the formation of petals and stamens [65]. It explains our observation that overexpression of *LlMADS37* substantially altered the morphogenesis of petals and stamens in transgenic *Arabidopsis* plants (Figure 5C). These identified CREs provide putative target sites for future bioengineering-based fine-tuning of the expression strength of the *LIMADS* genes of interest. Compared to directly editing gene exons, sequence modification in promoter regions can improve or repress the expression of product protein without changing its structure and function [66]. Thus, the findings of this study broaden our knowledge about the genomic background underlying the inflorescence and flower development of *L. littorea*, which can further facilitate artificial design of the relevant regulatory elements for the genetic improvement of *L. littorea* plants with high prolificacy.

Compared to Type II genes, our knowledge on the functions of Type I *MADS-box* genes is limited [67]. Several studies in *Arabidopsis* indicated that Type I genes participate in plant growth and reproduction, particularly in female gametophyte, embryo, and endosperm development [68,69]. Here, we found that one Type I gene (*LlMADS24*) was overexpressed in *L. littorea* flowers with stigma covered by petals (PC), while another two Type I genes (*LlMADS36* and *41*) were highly expressed in the flowers with uncovered stigma (PN). This suggests that Type I *LlMADS* genes also participate in the modulation of flower morphogenesis in *L. littorea*. Unlike most *LlMADS* genes, *LlMADS41* only consists of one SA-related CRE, and the promoter region of *LlMADS24* contains CREs associated with auxin and GA, indicative of special phytohormone regulation modes for the expression of these Type I genes. Moreover, three Type I genes (*LlMADS8*, *52*, and *55*) were significantly upregulated in leaves of *L. littorea* seedlings when exposed to cold stress (Figure 6A). These results provide new insights into the functions of Type I *MADS-box* genes in plant flower development and stress responses.

## 4. Materials and Methods

### 4.1. Identification of LlMADS Genes, Chromosomal Location, and Phylogenetic, Gene Structure, and Conserved Motif Analysis, as Well as Cis-Regulatory Element Prediction

Whole-genome sequences of *L. littorea* were obtained from the National Center for Biotechnology Information (NCBI) with the accession number GCA_024138015.1 [70], and MADS-box protein sequences of *Arabidopsis* were downloaded from the EnsemblPlants database (http://plants.ensembl.org/index.html/ (accessed on 28 June 2019)). Hidden Markov Model (HMM) libraries of the MADS domain (PF00319) and K domain (PF01486) were downloaded from the Pfam database (http://pfam.xfam.org/ (accessed on 3 February 2020)). HMMER3.0 was utilized to search for potential *MADS-box* genes in *L. littorea* with an e-value cutoff of 0.01 [71]. Physical locations, structures, and collinearity relationships of *LlMADS* genes were inferred using TBtools software (v2.154) [72]. Amino acid length, MW, and pI were assessed for the putative product proteins of each *LIMADS* gene of *L. littorea* using ExPASy Serve (http://www.expasy.org/ (accessed on 2 August 2024)). *LIMADS* genes were grouped into two clades, Type I and Type II, according to their similarities in amino acid sequences to *Arabidopsis*. For each clade, the phylogenetic relationships among the genes were inferred by a neighbor-joining (NJ) algorithm in MEGA v7.0 software [73] with a bootstrap analysis of 1000 replicates. The phylogenetic trees were visualized using the iTOL website (https://itol.embl.de/ (accessed on 15 August 2024)) [74]. Conserved motifs were predicted in each *LIMADS* gene using the MEME suite (https://meme-suite.org/meme/ (accessed on 18 August 2024)) with the number of motifs set to 10, and putative cis-regulatory elements in their promoter regions (2000 bp upstream the transcription start site) were scanned using the online web server PlantCARE (https://bioinformatics.psb.ugent.be/webtools/plantcare/html/ (accessed on 10 September 2024)) and visualized by TBtools software (v2.154).

### 4.2. Expression Characterization of LlMADS Genes in L. littorea Flowers and Functional Validation

Transcriptome data of two types of flowers of *L. littorea* (NCBI accession number: SRP127706) were employed to investigate the role of *LlMADS* genes during florescence of *L. littorea*. Differential expression analysis was performed between PC- and PN-type flowers using the DESeq2 package (v1.46.0) [75], where the genes with log_2_(fold-change) > 1 and adjusted *p*-value < 0.05 were considered upregulated upon stress exposure compared to CK, while those with log_2_(fold-change) < −1 and adjusted *p*-value < 0.05 were considered to be downregulated. WGCNA was then conducted to investigate the correlation of differentially expressed *LlMADS* genes to PC and PN phenotypes via the R package WGCNA (v1.72.1) [76]. Quantitative real-time PCR (qRT-PCR) was employed to assess the expression profiles of *LlMADS* genes across tissues. Samples of six tissues, including bud, perianth, stamen, pistil, seed, and leaf, were collected from three healthy *L. littorea* trees (biological replicates) in Tielu Bay, Sanya, Hainan, China (18°15′–18°17′ N, 109°42′–109°44′ E), immediately frozen with liquid nitrogen, and stored at −80 °C. Total RNA was extracted from each sample using the EASYspin Plus Plant RNA Kit (Aidlab, Beijing, China) following the manufacturer’s protocol, and cDNA was synthesized with the HiScript II 1st Strand cDNA Synthesis Kit (Vazyme, Nanjing, China). Gene-specific primers used for qRT-PCR were designed using Primer Premier 5.0 [77] (Table S2). RT-qPCR was conducted using SYBR^TM^ Select Master Mix (Thermo Fisher Scientific, Waltham, MA, USA) in a 20 μL reaction system, which contained 10 μL 2× qPCRmix, 0.2 μL forward primer, 0.2 μL reverse primer, 2 μL cDNA, and 7.6 μL double-distilled water. The reaction was carried out as follows in the StepOnePlus^TM^ Real-Time PCR System (Thermo Fisher Scientific, Waltham, MA, USA): 95 °C for 30 s, 30 cycles of 95 °C for 15 s, 60 °C for 15 s and 72 °C for 15 s, 95 °C for 30 s, 65 °C for 30 s, and 95 °C for 30 s. Each sample was run in three technical replicates. The *U6* gene was used as the internal reference for qRT-PCR. The expression levels were calculated using the 2^−△△Ct^ method and significance analyses were performed using GraphPad Prism 9 (GraphPad Software, Boston, MA, USA).

Transgenic lines of *A. thaliana* were generated to validate the function of *LlMADS37*, which displayed the highest correlation to the phenotype of PC compared to other differentially expressed *LlMADS* genes. Briefly, the full-length coding sequences (CDSs) of *LlMADS37* were amplified by PCR with the specific primers listed in Table S2, cloned into the pBinGlyRed vector, and then transferred into *A. thaliana* using the floral dip method via Agrobacterium tumefaciens GV3101. The seeds of the positive transgenic plants were screened on the MS agar medium containing 20 mg/L Hygromycin after vernalization, and the seedlings were transplanted into the soil. The seeds and seedings of *A. thaliana* were cultivated in a greenhouse with 22 °C, 40–50% humidity, and 16 h light/8 h darkness. The genomic DNA for each plant was extracted using Takara DNAiso Reagent (Kusatsu, Shiga, Japan), and the target gene was detected with PCR. Homozygote T3 generation plants, which are 100% resistant to hygromycin, were used for phenotype analysis. The qRT-PCR method was used to evaluate the expression level of *LlMADS37* in *OX-LlMADS37 A. thaliana* lines. The *U6* gene of *A. thaliana* (*AT5G51170*) was used as the internal reference for expression normalization. The Wilcoxon test was employed to examine the statistical significance for the difference in *LlMADS37* expression and the period from germination to flowering between WT and each *OX-LlMADS37* transgenic line.

### 4.3. Comparative Transcriptome Analysis of LlMADS Gene in Leaves in Response to Salt and Cold Stresses

To investigate the functions of *LlMADS* genes in responding to abiotic stress, comparative transcriptome analyses were performed to assess their changes in expression levels under salinity and cold stress. In particular, two groups of one-year-old *L. littorea* seedlings with similar growth conditions were selected and treated with fresh water (CK) and 400 mmol·L^−1^ NaCl (salt stress), respectively, for 3 days. Total RNA was extracted from leaves of each treated individual using an EASYspin Plus Complex Plant RNA Kit (Aidlab Biotechnologies Co., Ltd., Beijing, China). For the treatment group, three biological replicates were employed. The transcriptome of each sample was sequenced on an Illumina Novaseq 6000 platform (Illumina Inc., San Diego, CA, USA). For cold stress, the transcriptome data from our previous study (NCBI accession number: SRP352309) were reused for the analysis. For each sample, the quality of raw sequencing reads was evaluated using FastQC (v0.11.9), and data cleaning was carried out following the procedures described in our previous studies [43]. The high-quality reads were aligned to the reference genome of *L. littorea* [70] using HISTA2 (v2.2.1) [78], and gene expression levels were quantified by the abundance of reads aligned to the genes using featureCounts of the Subread software (v2.0.2) [79]. For both stress scenarios, differential expression analysis was performed between the stress condition and CK, as described above. For the cold stress condition, WGCNA was conducted to investigate the role of *LlMADS* genes in gene co-expression networks, and the functional importance of genes in the modules of interest was then assessed by Gene Ontology (GO) enrichment analysis using the clusterProfiler package (v4.6.0) [80] with a *p*-value cutoff of 0.05.

## 5. Conclusions

In this study, we identified 63 *MADS-box* genes in the genome of *L. littorea* and comprehensively characterized their features, including genomic locations, gene and protein structures, phylogenetic relationships, origin, and CREs. Comparative expression analysis and transgenic experiments further showed that some *LlMADS* genes are associated with flower morphogenesis and the response to cold stress in *L. littorea*. These findings highlight the biological importance of *MADS-box* genes in regulating the reproduction and stress acclimation of *L. littorea* and provide putative targets for future genetic improvement of the prolificacy of *L. littorea* to protect and restore this endangered mangrove species.

## Data Availability

The transcriptome sequencing data were deposited in the National Center for Biotechnology Information (NCBI) Gene Expression Omnibus (GEO) with the accession number: GSE283552.

## Supplementary Materials

The supporting information can be downloaded at https://www.mdpi.com/article/10.3390/ijms26041680/s1.
