# Supplementary material for: Genome-Wide Investigation of MADS-Box Genes in Flower Development and Environmental Acclimation of Lumnitzera littorea (Jack) Voigt
Source: Int J Mol Sci. 2025 Feb 16;26(4):1680. doi: 10.3390/ijms26041680 (PMC11855919; doi:10.3390/ijms26041680)
Supplement: Supplementary file 1 [file ijms-26-01680-s001.zip › ijms-3404422-supplementary.pdf]

Table S1. Features of *LlMADS* genes

Gene ID	Locus ID in genome	Chr	Strand	AA_length	pI	MW(KDa)	type	pfam
LlMADS1	evm.model.78.136	1	-	409	7.78	45.92	type I	PF00319
LlMADS2	evm.model.64453.514	1	-	214	8.89	24.10	type II	PF00319, PF01486
LlMADS3	evm.model.5.364	2	+	214	5.11	24.14	type II	PF00319
LlMADS4	evm.model.8.136	2	+	377	6.37	42.84	type I	PF00319
LlMADS5	evm.model.96.195	2	-	216	9.23	24.73	type I	PF00319
LlMADS6	evm.model.64482.246	2	-	231	10.13	26.67	type II	PF00319, PF01486
LlMADS7	evm.model.98.150	2	+	267	5.15	29.94	type I	PF00319
LlMADS8	evm.model.98.152	2	+	293	4.26	32.59	type I	PF00319
LlMADS9	evm.model.98.231	2	+	202	6.64	22.89	type I	PF00319
LlMADS10	evm.model.32.392	2	-	246	9.54	27.93	type II	PF00319, PF01486
LlMADS11	evm.model.7.87	3	+	226	9.31	26.58	type II	PF00319, PF01486
LlMADS12	evm.model.7.597	3	+	270	8.06	30.23	type II	PF00319, PF01486
LlMADS13	evm.model.7.1179	3	+	392	5.33	43.28	type I	PF00319
LlMADS14	evm.model.64489.14	3	-	267	8.05	30.39	type II	PF00319, PF01486
LlMADS15	evm.model.64427.217	3	-	174	9.85	19.71	type I	PF00319
LlMADS16	evm.model.77.18	4	-	216	8.59	24.87	type I	PF00319
LlMADS17	evm.model.71.43	4	-	231	9.79	26.86	type I	PF00319
LlMADS18	evm.model.64468.254	4	-	217	6.75	24.65	type II	PF00319, PF01486
LlMADS19	evm.model.57.82	4	-	363	6.9	41.17	type II	PF00319
LlMADS20	evm.model.1.393	5	+	256	9.41	29.23	type I	PF00319
LlMADS21	evm.model.1.264	5	-	246	8.59	28.04	type II	PF00319, PF01486
LlMADS22	evm.model.59.3	5	+	243	8.86	27.74	type II	PF00319, PF01486
LlMADS23	evm.model.37.67	5	-	161	9.9	18.67	type I	PF00319
LlMADS24	evm.model.99.279	5	-	412	6.54	46.65	type I	PF00319
LlMADS25	evm.model.64481.35	6	+	249	8.57	28.49	type II	PF00319, PF01486
LlMADS26	evm.model.14.1042	6	-	243	8.81	27.93	type II	PF00319, PF01486
LlMADS27	evm.model.64419.2	8	+	149	9.25	17.36	type II	PF00319, PF01486
LlMADS28	evm.model.64419.1	8	-	234	8.99	27.07	type II	PF00319, PF01486
LlMADS29	evm.model.11.362	8	+	285	6.55	33.32	type II	PF00319, PF01486
LlMADS30	evm.model.80.56	8	+	251	8.69	28.54	type II	PF00319, PF01486
LlMADS31	evm.model.4.347	9	-	333	4.93	35.79	type I	PF00319
LlMADS32	evm.model.64302.39	9	-	161	10.13	18.62	type I	PF00319
LlMADS33	evm.model.76.192	9	-	155	9.91	17.92	type II	PF00319, PF01486
LlMADS34	evm.model.64440.66	9	+	103	10.63	12.11	type II	PF00319
LlMADS35	evm.model.81.131	9	-	167	9.9	19.41	type II	PF00319, PF01486
LlMADS36	evm.model.6.767	10	+	162	8.65	18.74	type I	PF00319
LlMADS37	evm.model.6.807	10	-	227	9.2	26.67	type II	PF00319, PF01486
LlMADS38	evm.model.64469.127	10	-	215	6.6	24.72	type I	PF00319
LlMADS39	evm.model.9.30	11	+	142	9.85	16.57	type II	PF00319, PF01486
LlMADS40	evm.model.9.33	11	+	222	8.81	25.70	type II	PF00319, PF01486
LlMADS41	evm.model.9.203	11	+	340	9.31	38.65	type I	PF00319

LlMADS42	evm.model.9.204	11	+	313	6.4	35.82	type I	PF00319
LlMADS43	evm.model.9.205	11	+	298	7.11	34.35	type I	PF00319
LlMADS44	evm.model.9.249	11	-	234	8.94	26.04	type I	PF00319
LlMADS45	evm.model.9.250	11	-	213	6.11	24.70	type I	PF00319
LlMADS46	evm.model.9.251	11	-	237	9.86	26.62	type I	PF00319
LlMADS47	evm.model.9.252	11	-	240	9.08	27.23	type I	PF00319
LlMADS48	evm.model.64477.219	11	-	171	9.6	19.98	type II	PF00319, PF01486
LlMADS49	evm.model.64254.9	11	+	167	8.56	18.71	type I	PF00319
LlMADS50	evm.model.64464.116	11	-	186	8.86	21.21	type II	PF00319, PF01486
LlMADS51	evm.model.40.343	11	+	204	9.38	23.24	type II	PF00319, PF01486
LlMADS52	evm.model.2.494	11	-	260	5.08	29.47	type I	PF00319
LlMADS53	evm.model.2.495	11	-	258	4.88	28.91	type I	PF00319
LlMADS54	evm.model.2.496	11	-	258	4.93	29.10	type I	PF00319
LlMADS55	evm.model.2.497	11	-	223	4.82	25.01	type I	PF00319
LlMADS56	evm.model.39.19	11	+	200	9.61	22.75	type I	PF00319
LlMADS57	evm.model.39.14	11	+	282	9.3	31.23	type I	PF00319
LlMADS58	evm.model.3.1210	12	-	234	9.35	27.01	type II	PF00319, PF01486
LlMADS59	evm.model.3.1246	12	-	250	7.63	28.34	type II	PF00319, PF01486
LlMADS60	evm.model.64455.124	12	-	319	5.32	36.30	type II	PF00319
LlMADS61	evm.model.42.185	12	+	253	9.14	28.88	type II	PF00319, PF01486
LlMADS62	evm.model.64485.19	12	+	164	9.18	19.32	type II	PF00319, PF01486
LlMADS63	evm.model.64485.170	12	+	226	8.72	26.57	type II	PF00319, PF01486

MW, molecular weight. pI, isoelectric point.

Table S2. Sequences of PCR primers used in this study

Primers	Primer sequences（5'-3')
LlMADS2-F	TCCTCCAGGATGCAAGAC
LlMADS2-R	CTCCCTTTATCCGTCTCA
LlMADS3-F	CAATGAGTTATCGGTGTTAT
LlMADS3-R	TCTTTATCTCCAATGTCCC
LlMADS14-F	GGGTAGAGGAAGAGGGA
LlMADS14-R	GCACTAGAGTATTCATAAAGC
LlMADS18-F	GAGGATGCCCAGAGTAA
LlMADS18-R	ATTCAAGCAAGCCAAAT
LlMADS19-F	GTTTGCTCAATTAACTCCA
LlMADS19-R	AGTCTTCCATCTGCCTAA
LlMADS24-F	CGGACAACATACGAGAAA
LlMADS24-R	TCACTACCATCAGGACCAT
LlMADS35-F	AAGTCACCTTCTCCAAACGT
LlMADS35-R	TCTTCTCCTCTCATCTGCCT
LlMADS36-F	GAGATAAGTGCCCTATGC
LlMADS36-R	CGAGCCTTACTGATTCTAC
LlMADS37-F	TGGGTCGTGGTAAGATTGAG
LlMADS37-R	ACCTTAGCATCGCAAAGA
LlMADS41-F	GAGTGCAAGAACAACAACCA
LlMADS41-R	AACGTCTGAGAGCCCAAAA
LlMADS51-F	AAGGCTACCCTTGGTCC
LlMADS51-R	GGGCACTGATTTCGTTC
LlMADS63-F	ATCTTTGGAGCACTCATTA
LlMADS63-R	TTTTCCTTGTATCAATCTGA
LlMADS37-FP	GGAATTCATGGGTCGTGGTAAGATTGAGAT
LlMADS37-RP	TCCCCCCGGGTTAATCGAGGCGCATATCGG
U6-F	ACATCCGATAAAATTGGAACGA
U6-R	TTTTTTTGGACCATTTCTCGAT

Table S3. *LlMADS* genes positively correlated with the phenotype of PC

Genes	Correlation coefficients
LlMADS11	0.94
LlMADS18	0.95
LlMADS25	0.87
LlMADS28	0.93
LlMADS37	0.98

Table S4. *LlMADS* genes positively correlated with the phenotype of PN

Genes	Correlation coefficients
LlMADS19	0.94
LlMADS36	0.89

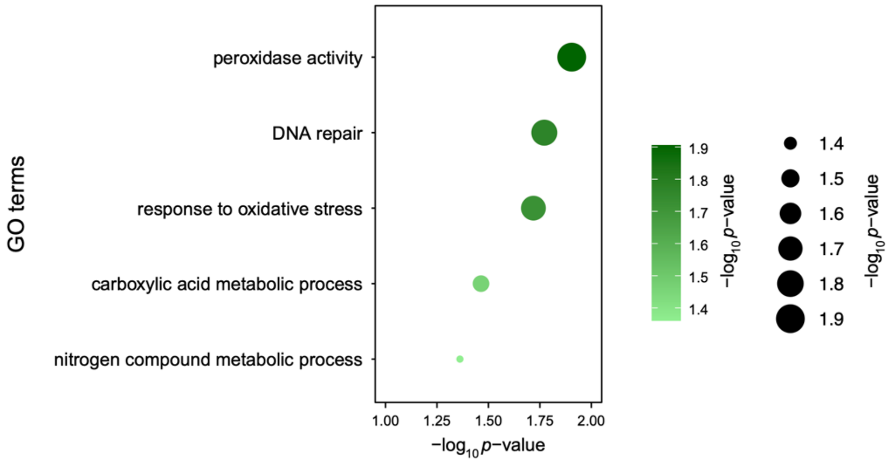

Figure S1. Representative Gene Ontology (GO) terms enriched for the genes of the "green" module, which is positive correlated with the response of *L. littorea* to chilling stress.
